# Ischemic Stroke as the Initial Presentation of Sickle Beta Thalassemia in an Adolescent: A Case Report

**DOI:** 10.7759/cureus.84254

**Published:** 2025-05-16

**Authors:** Shivangi Sinha, Jignesh Sharma, Sabavath Arun, Amber Kumar, Shikha Malik

**Affiliations:** 1 Pediatric Medicine, All India Institute of Medical Sciences, Bhopal, Bhopal, IND

**Keywords:** adolescent, beta thalassemia, exchange transfusion, genetic screening, sickle cell disease, stroke

## Abstract

Ischemic stroke in children is rare and often signifies an underlying systemic disorder. We report the case of a 13-year-old girl presenting with sudden-onset right-sided weakness, facial deviation, and aphasia. Neuroimaging revealed a left fronto-parietal infarct with hemorrhagic transformation and left middle cerebral artery thrombosis. Peripheral smear showed sickle cells; high-performance liquid chromatography indicated sickle beta thalassemia (HbS 76%, HbA2 5.3%, HbA 7.7%). Genetic analysis confirmed compound heterozygosity: HBB: c.20A>T (codon 6 A>T, sickle cell mutation) and HBB: c.92+5G>C (IVS 1-5 G>C, beta thalassemia mutation). She was managed with exchange transfusions and initiated on hydroxyurea. Family screening revealed her father was a sickle trait carrier, her mother a beta-thalassemia carrier, and her asymptomatic brother had the same compound heterozygosity. Arterial stroke can be an atypical and rare presentation of sickle beta thalassemia, highlighting the quiet progression of hemoglobinopathies and the necessity for heightened clinical suspicion. Early diagnosis through family screening and timely intervention can prevent catastrophic complications in at-risk individuals.

## Introduction

Sickle cell disease (SCD) and beta-thalassemia are among the most prevalent inherited hemoglobin disorders globally, with a significant burden in India and other regions of the Indian subcontinent [1,2]. SCD and beta-thalassemia in a compound heterozygous state, I.e, sickle beta-thalassemia, is marked by wide phenotypic variability [3]. The clinical course can range from asymptomatic states to severe hemolytic anemia and vaso-occlusive crises, depending on the specific beta globin gene mutation and hemoglobin S (HbS) level [4].

Ischemic stroke is a recognized complication in homozygous SCD (HbSS), particularly during childhood, but it is exceptionally rare as a presenting feature in sickle beta-thalassemia. This diagnostic challenge is further compounded in regions without universal screening, where atypical presentations may delay appropriate intervention.

In this report, we describe a rare case of an adolescent girl presenting with arterial stroke as the first manifestation of sickle beta thalassemia. This highlights the importance of maintaining a high degree of clinical suspicion, providing timely exchange transfusion, early genetic testing, and family screening in ensuring early diagnosis and prevention of severe complications.

## Case presentation

A 13-year-old female child, firstborn of a non-consanguineous marriage, with no significant past and family history, presented with sudden onset right-sided hemiparesis, left-sided deviation of the angle of the mouth, and inability to speak ten days before hospital admission. There was no prior history of trauma, seizures, fever, blood transfusion, or known hematological illness.

On examination, she was conscious, afebrile, and normotensive. The neurological assessment revealed right-sided hemiparesis (Grade 3/5), upper motor neuron facial palsy, and Broca’s aphasia. The rest of the systemic examination was unremarkable. Her National Institutes of Health Stroke Scale (NIHSS) score was 17, suggestive of a moderate stroke.

Initial hematological investigations showed mild anemia with hemoglobin of 9.2 mg/dL, and peripheral smear suggested microcytic hypochromic red cells with occasional sickled cells. The laboratory test results are given in Table 1. High-performance liquid chromatography (HPLC) was indicative of a compound heterozygous for beta-thalassemia and HbS (HbS-76%, HbA2-5.3%, HbA-7.7%), and other investigations, such as serum homocysteine, iron profile, and coagulation profile, were normal. 

**Table 1:** Laboratory parameters of the index patient ANA: anti-nuclear antibodies; ANCA: antineutrophil cytoplasmic antibody; PT: prothrombin time; INR: international normalised ratio; aPTT: activated partial thromboplastin time

Parameters	Patient values	Reference ranges
Hemoglobin	9.2 gm/dl	11-15 gm/dL
Total leucocyte count	10690/microL	4000-11000/microL
Platelet Count	4.15lakh/microL	1.5-4.5lakh/microL
Bilirubin Total/Direct/Indirect	1.40/0.31/1.09 mg/dL	
Aspartate Transaminase	72 U/L	<35
Alanine Aminotransferase	18 U/L	<35
Alkaline phosphatase	146.U/L	30-120
Urea/Creatinine	21mg/dL / 0.5mg/dL	20-40mg/dL / <1mg/dL
Serum Iron	92mcg/dL	50-150mcg/dL
Homocysteine	7nmol/mL	4.3-11.4nmol/mL
Lupus anticoagulant	Negative	
ANA	Negative	
ANCA	Negative	
PT/INR value	12 second	11-13.5 seconds
aPTT	29 second	25-35 seconds

**Figure 1:**
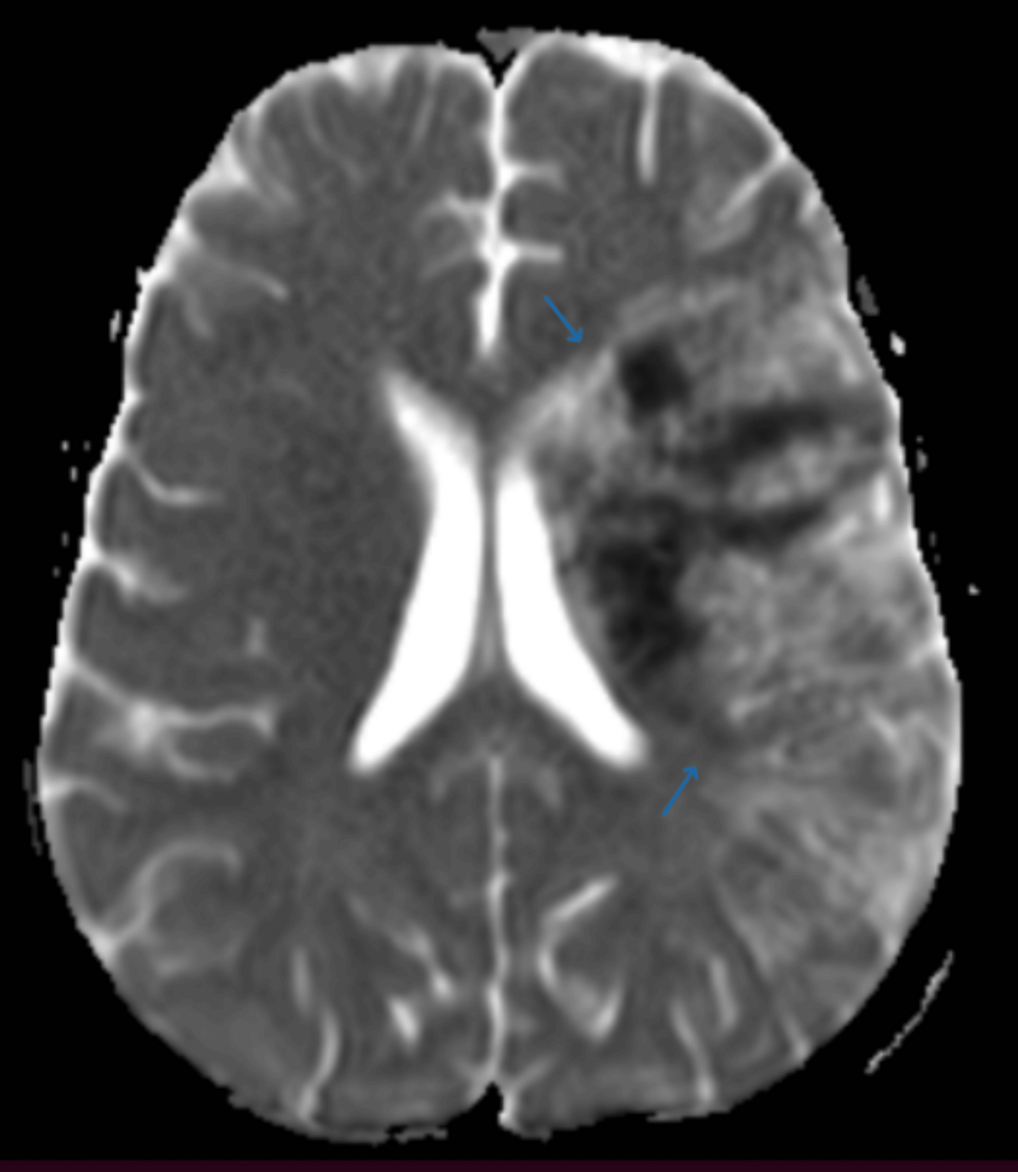
Axial T2-weighted MRI brain showing hyperintense signal in the left frontoparietal cortex and subcortical white matter, consistent with acute infarction involving the left MCA territory. MCA: middle cerebral artery

**Figure 2:**
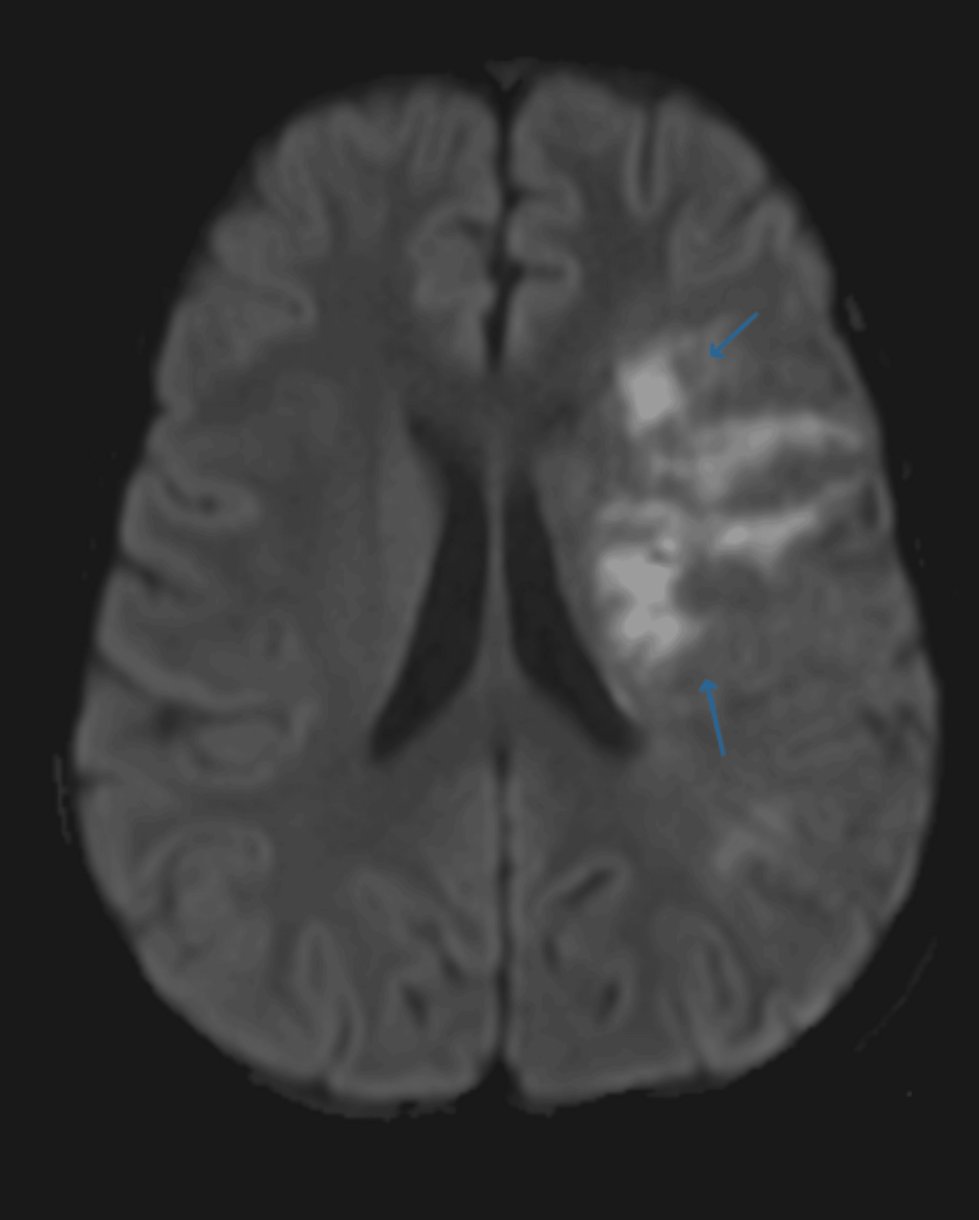
Diffusion-weighted imaging (DWI) showing restricted diffusion in the left frontoparietal region, confirming acute ischemic infarct.

**Figure 3:**
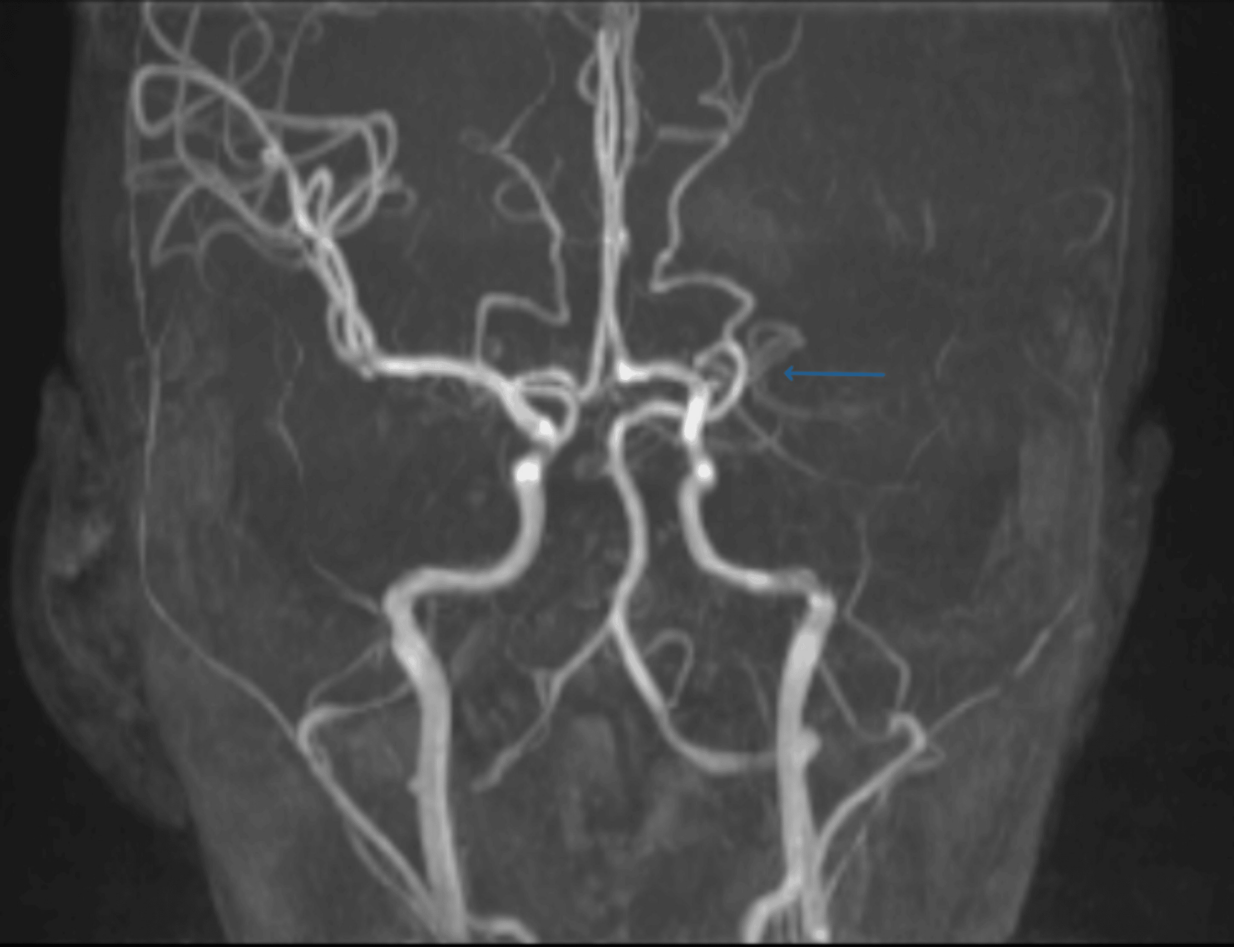
MRA showing attenuation and narrowing of the left MCA, suggestive of large-vessel arteriopathy secondary to sickle beta-thalassemia MRA: magnetic resonance angiography; MCA: middle cerebral artery

Given the acute neurological deficit, an MRI brain was performed, which showed an acute infarction in the left frontoparietal region with cortical and subcortical involvement (Figures 1-2). The lesion exhibited hyperintensity on diffusion-weighted imaging (DWI) and hypointensity on apparent diffusion coefficient (ADC), consistent with acute ischemic stroke. Magnetic resonance angiography (MRA) of the cerebral vessels revealed narrowing of the left middle cerebral artery (MCA) (Figure 3), indicating large-vessel arteriopathy.

Genetic analysis revealed the patient to be compound heterozygous for HbS and Hb β+ gene, confirming sickle beta-thalassemia. Specifically, the patient had HBB: c.92+5G>C (IVS 1-5 G>C) mutation indicative of beta-thalassemia trait and HBB: codon 6 A>T mutation indicative of sickle cell trait, confirming sickle beta-thalassemia (Table 1). Her father was a thalassemia carrier, and her mother was a sickle carrier. Her asymptomatic brother carried the same compound heterozygous mutation, and hence, he was initiated on hydroxyurea.

**Table 2:** HPLC and genetic mutation details HPLC: high-performance liquid chromatography; M: male; F: female

Individuals tested, relationship (age/sex)	HPLC	Mutation Details	Zygosity	Interpretation
Index Case (13/F)	HbA- 7.7%, HbA2- 5.3% HbS- 76%, HbF- 11%	HBB: c.92+5G>C (IVS 1-5 G>C) + HBB: codon 6 A>T	Compound Heterozygous	Sickle Beta+ Thalassemia
Sibling (10/M)	HbA-6.7%, HbA2-6.2% HbS- 76.6%, HbF- 8.5%	HBB: c.92+5G>C (IVS 1-5 G>C) + HBB: codon 6 A>T	Compound Heterozygous	Sickle Beta+ Thalassemia
Father (33/M)	HbA-83.2% HbA2- 5.9% HbS- nil , HbF- <0.8	HBB: c.92+5G>C (IVS 1-5 G>C)	Heterozygous	Beta Thalassemia Trait
Mother (30/F)	HbA- 52.2% HbA2- 3.5% HbS-37.2%, HbF- <0.8%	HBB: codon 6 A>T	Heterozygous	Sickle Cell Trait

She was treated with two cycles of exchange transfusion to lower the HBS concentration to below 30%. Hydroxyurea was started, and neurologic rehabilitation was initiated. The patient showed gradual improvement in motor power (NIHSS score 8) and was discharged with follow-up plans for transfusion and stroke prevention.

## Discussion

Inherited hemoglobin disorders, including SCD and beta-thalassemia, cause chronic hemolytic anemia due to a reduced lifespan of red blood cells resulting from abnormalities in hemoglobin structure and/or globin chain production [5]. Changes in hemoglobin physiology can create a pro-thrombotic environment, making patients more susceptible to both venous and arterial thrombosis through mechanisms such as endothelial dysfunction, increased blood viscosity, and chronic inflammation. In patients with SCD, the cumulative incidence of venous thrombosis is about 11.3%, and cerebrovascular accidents (strokes) impact about 4% of cases [6]. While in beta-thalassemia, thromboembolisms are observed in around 1-5% of patients [7].

Silent brain infarctions, which are only detected by neuroimaging, are common, with a reported prevalence of 27-60% in beta-thalassemia intermedia and 21.6% in children with SCD aged 6-19 years [8,9]. These incidents demonstrate the silent development of thrombo-embolic events in hemoglobinopathies and frequently occur before clinically obvious strokes. The vascular risk associated with unstable hemoglobinopathies is further reinforced by the association of rare hemoglobin variations (such as Hb Taybe, Hb Hiroshima, and Hb Madrid) with arterial thrombotic events [10].

Compared to adult rates, the estimated global incidence of pediatric stroke is minimal, at two per 100,000 children [11]. Because of this rarity, stroke in children requires a careful investigation for underlying causes, such as hemoglobinopathies. The fact that arterial stroke is the initial symptom of sickle beta-thalassemia in this instance highlights how these conditions are silent but progressing. The diagnosis was delayed due to the lack of organomegaly, previous transfusions, or known family history, highlighting the fact that hemoglobinopathies might go undiagnosed until a significant event, such as a stroke, occurs.

Sickled red blood cells cause vascular occlusion by endothelial adhesion, hemolysis, inflammation, and a hypercoagulable state, which is part of the pathogenesis of stroke in SCD. These processes lead to vaso-occlusion and ischemia, which are exacerbated by low nitric oxide bioavailability and elevated blood viscosity [8,12]. These concerns might continue in compound heterozygous conditions such as sickle beta thalassemia, particularly if the beta thalassemia mutation allows for moderate HbS levels, as in the case of the current patient.

Crucially, this cerebrovascular incident revealed a concealed familial burden of disease in addition to prompting the diagnosis of the index patient. Both of the parents were found to be carriers of sickle cell and beta thalassemia traits by genetic testing, and the sibling was likewise compound heterozygous but asymptomatic. Hydroxyurea therapy was also started for the sibling as a proactive intervention strategy to prevent future complications [13]. This case emphasizes the essential role of family screening and prompt therapeutic intervention, especially in regions where hemoglobinopathies are common but frequently overlooked. By detecting at-risk individuals before they experience irreversible complications, healthcare providers can start disease-modifying treatments and offer genetic counseling, thereby influencing the disease's progression and improving long-term results [14,15].

The take-home message of this reported case is: (i) Pediatric stroke may be the first sign of underlying hemoglobinopathy; (ii) Early detection and treatment, especially exchange transfusion, are essential for reducing the risk of further strokes and enhancing therapeutic outcomes in sickle beta-thalassemia; (iii) Genetic counseling plays a vital role for families with hereditary hemoglobinopathies since it helps avoid future recurrence and enables early diagnosis in unaffected relatives.

## Conclusions

A 13-year-old girl who had an ischemic stroke as her first manifestation of sickle beta-thalassemia serves as a reminder of the importance of increased clinical suspicion in young people with unusual neurological symptoms. Acute catastrophic presentation in the form of right-sided hemiparesis, facial nerve palsy, and aphasia without any significant past and family history needed urgent neuroimaging, which showed left middle cerebral artery thrombosis. Further, hematological and genetic investigations confirmed compound heterozygosity for SCD (HBB: c.20A>T) and beta-thalassemia (HBB: c.92+5G>C) mutations. Family screening further revealed the undiagnosed familial burden. Her asymptomatic brother carried the same compound heterozygosity, while her parents were carriers of sickle cell and beta-thalassemia traits. 

Early institution of exchange transfusions and hydroxyurea resulted in a marked reduction in her HbS levels. The patient showed significant clinical improvement, which was evidenced by her NIHSS score of 8 at the time of discharge compared to her admission score of 17. Timely medical diagnosis and therapeutic intervention, along with neurological rehabilitation, are the cornerstone in reducing stroke-related morbidity in sickle beta thalassemia. The case's key clinical insight is that, especially in areas without universal screening, hemoglobinopathies, particularly sickle beta-thalassemia, can manifest with life-threatening complications like arterial stroke, even in the absence of previous symptoms or known family history. A multidisciplinary approach, including a hematologist, geneticist, and neurologist, is essential to improve long-term outcomes for these individuals and their families.
